# Molecular epidemiology of antimicrobial resistance and virulence of carbapenem-resistant *Klebsiella pneumoniae* in South China

**DOI:** 10.3389/fmicb.2026.1817833

**Published:** 2026-04-16

**Authors:** Yuting He, Ruting Deng, Wenbin Lin, Xiaoya Yang, Yao Li, Yaoxiang Yang, Cha Chen, Bin Huang

**Affiliations:** 1 Department of Laboratory Medicine, The First Affiliated Hospital, Sun Yat-sen University, Guangzhou, China; 2 Department of Laboratory Medicine, Guangdong Provincial Hospital of Traditional Chinese Medicine, Guangzhou, Guangdong, China

**Keywords:** antimicrobial resistance, carbapenem-resistant *Klebsiella pneumoniae*, hvCRKP, resistance mutations, virulence

## Abstract

**Objective:**

Carbapenem-resistant *Klebsiella pneumoniae* (CRKP) has become a worldwide public health concern due to its high morbidity and mortality rate. A retrospective study was conducted to explore the molecular epidemiology of antimicrobial resistance and virulence of CRKP in South China.

**Methods:**

108 CRKP isolates were collected from multiple hospitals in South China. The characteristics of the CRKP strains, including antimicrobial resistance genes, mutations and virulence factors, were analyzed using whole-genome sequencing and the software Kleborate. A phylogenetic tree was constructed to illustrate evolutionary diversification.

**Results:**

The main Sequence Type (ST) type, capsular serotype, and LPS serotype among CRKP isolates in South China were ST11, KL47, and OL101, respectively. KPC-2 was the most prevalent carbapenemase type. Notably, KPC-12, a recently identified and rarely reported variant of KPC-2, was discovered in four CRKP strains. All ST11 CRKP isolates harbored porin and efflux pump regulator mutations of AcrR-43%, OmpK35-17%, and OmpK36GD as well as fluoroquinolone mutations of GyrA-83I, GyrA-87G, and ParC-80I. All CRKP strains exhibited complex multi-drug resistance (MDR) phenotypes. The most common types of aerobactin (*iuc*) and yersiniabactin (*ybt*) were *iuc1* and *ybt9* ICEKp3, respectively. There were 17 hvCRKP (hypervirulent carbapenem-resistant *Klebsiella pneumoniae*) strains found in our study, which were mainly ST11-KL64 and ST413-1LV-KL112, harboring the *bla*_KPC-2_ gene and *bla*_NDM-1_ gene, respectively.

**Conclusion:**

In South China, highly virulent and MDR CRKP strains show a trend toward epidemic, underscoring the urgent need for ongoing genomic surveillance and stringent infection control measures. KPC-2 variant KPC-12 and ST413-1LV-KL112 hvCRKP, a novel type of hvCRKP carrying *bla*_NDM-1_, require particular attention and further investigation.

## Introduction

*Klebsiella pneumoniae* is a Gram-negative opportunistic pathogen that has gained increasing attention for its multidrug resistance, high virulence, and high transmissibility (Lei et al., 2024). *K. pneumoniae* can cause various types of infections including pneumonia, soft tissue and surgical wound infections, bloodstream infections, and intra-abdominal infections, which can be life-threatening and pose a great challenge to public health. *K. pneumoniae* infections account for nearly 10% of nosocomial bacterial infections, with a mortality rate of about 50% in *K. pneumoniae*-caused pneumonia (Karampatakis et al., 2023). Gradually, *K. pneumoniae* has evolved from classic *K. pneumoniae* into carbapenem-resistant *K. pneumoniae* (CRKP) through the acquisition of carbapenem resistance genes and into hypervirulent *K. pneumoniae* (hvKp) through the acquisition of virulence factors (Han et al., 2022). Following the emergence of extended-spectrum β-lactamase-producing *K. pneumoniae* strains, CRKP has become a worldwide public health concern due to its high morbidity and mortality rate (Lin et al., 2024). The estimated mortality rate related to CRKP infections ranges from 33.1 to 41.4%, and the prevalence of CRKP has increased to 49% in China according to epidemiological data from surveillance systems (Agyeman et al., 2020; Wang et al., 2024). Meanwhile, CRKP is often associated with complex multi-drug resistance (MDR) phenotypes, posing hurdles for clinical therapy (Di Pilato et al., 2024). HvKp could cause life-threatening community-acquired infections such as liver abscess, pneumonia, meningitis, and endophthalmitis in younger healthy populations (Dai and Hu, 2022). In recent years, HvKp and CRKP have evolved into hypervirulent carbapenem-resistant *Klebsiella pneumoniae* (hvCRKP) through the convergence of hypervirulent and carbapenem-resistance plasmids (Chen et al., 2025). The virulence factors of *K. pneumoniae* include four main classes: capsule, lipopolysaccharide, siderophores, and fimbriae (Mendes et al., 2023). The virulence of hvKp isolates is mainly related to mucoid regulators and siderophores (Jiang et al., 2025). Mucoid regulators include *RmpA* and *RmpA2*, which could strengthen capsule production and mucoviscosity. The siderophores secreted by *K. pneumoniae*, including plasmid-encoded aerobactin (*iuc*) and salmochelin (*iro*), as well as chromosomally encoded yersiniabactin (*ybt*) and colibactin (*clb*), mainly facilitate iron acquisition. It was reported that siderophores were more prevalent in hvKp than classic *K. pneumoniae*, and *iuc* was one of the most important virulence determinants of hvKp (Zhou et al., 2023). The most important mechanism underlying carbapenem resistance in CRKP is the production of carbapenemases including KPC, NDM, OXA-48-like, IMP, and VIM. In China, the predominant carbapenemase type is KPC-2 and the dominant sequence type (ST) is ST11 (Shi et al., 2024). KPC variants are defined as the substitution, insertion, or deletion of amino acid sequences compared to the wild-type *bla*_KPC_ sequence. More than 150 *bla*_KPC_ variants have been discovered worldwide so far, posing new threats to global public health (Ding et al., 2023). In a systematic and comprehensive global genomic analysis that included 35,471 CRKP isolates collected from 101 countries during 1996–2023, 1,046 known ST carrying different kinds of carbapenemase genes have been identified (Shi et al., 2026). However, regional epidemiology can evolve and there are discrepancies between regions. To understand predominant carbapenemase types and STs globally, exploring regional-specific carbapenemase variants and STs carrying different kinds of carbapenemase genes could inform targeted infection prevention measures and regional surveillance networks, provide insights into local evolutionary pathways, and enable risk stratification and targeted monitoring of high-risk populations. With the rapid development and application of whole genome sequencing (WGS) technology and bioinformatics, studying the molecular epidemiology of pathogenic strains has become increasingly feasible. In our study, CRKP strains from multiple hospitals in South China were collected. By using whole genome sequencing and the Kleborate software, the molecular characteristics of those strains, including antimicrobial resistance genes, mutations, and virulence genes, were analyzed. Understanding the potential regional CRKP epidemiological features, especially the hvCRKP and KPC variants, provides a theoretical basis for optimizing antibiotic administration and controlling the spread of resistant strains in a timely manner.

## Materials and methods

### Bacterial isolates

Between 2018 and 2021, 108 CRKP isolates were collected from several hospitals in South China. All isolates were identified as *K. pneumoniae* by MALDI-TOF MS (bioMérieux, France). Antimicrobial susceptibility testing was performed using the VITEK-2 COMPACT system (bioMérieux, France), and the results were further confirmed by the broth microdilution method. The breakpoints for carbapenem antibiotics as reported in the 35th edition of the M100 document of the Clinical and Laboratory Standards Institute (CLSI) were adopted for CRKP isolates. CRKP was defined as resistant to at least one carbapenem, including imipenem, meropenem, and ertapenem. The minimal inhibitory concentration (MIC) for imipenem or meropenem ≥4 mg/L or ertapenem ≥2 mg/L was defined as resistance. CRKP isolates were classified as hvCRKP or non-hvCRKP based on the two following criteria: (i) *RmpA*/*RmpA2*-positive and (ii) *iuc*-positive (Lan et al., 2021; Qala Nou et al., 2025). The isolates were preserved in LB broth with 40% glycerol and stored at −80 °C until being processed for DNA extraction.

### Whole-genome sequencing

Bacterial genomic DNA was extracted using the MiniBEST DNA Extraction Kit (Takara, Bio Inc.) according to the manufacturer’s instructions. According to the manufacturer’s instructions. Sequencing libraries were prepared using the QIAseq FX DNA Library Kit (QIAGEN, Germany). Qubit 2.0 (Thermo, USA) was used to conduct preliminary quantification. Whole-genome sequencing was performed on the Illumina NextSeq 500 platform (Illumina, USA). Fastq v0.21.0 was used to trim the adaptor, unreliable reads, and low-quality bases of raw sequencing reads. Paired reads were assembled by SPAdes v3.13.0 and all genomes were annotated by Prokka v1.14.6.

### Kleborate analysis

All *K. pneumoniae* genomes were analyzed using the software Kleborate.^1^ Kleborate was primarily developed to screen genome assemblies of *K. pneumoniae* for species; multi-locus sequence typing (MLST); ICEKp-associated virulence loci including *ybt*, *clb*, *iro*, and hypermucoidy (*rmp*); and virulence plasmid-associated loci including *iro*, *iuc*, hypermucoidy (*rmp*, *rmpA2*), K (capsule), and O antigen (LPS) serotype prediction. It also identifies antimicrobial resistance determinants including acquired antimicrobial resistance (AMR) genes, chromosomal-encoded AMR mutations involving fluoroquinolone-resistance mutations in GyrA (positions 83 and 87) and ParC (positions 80 and 84), SHV β-lactamase mutations, and mutations of OmpK35/OmpK36 porins and efflux pump regulators (AcrR, RamA, RamR, and RomA) associated with reduced sensitivity to β-lactamases.

### Phylogenetic tree construction

The phylogenetic analysis utilizes the concatenated core gene alignment. The core genes are extracted using the Up-to-date Bacterial Core Gene (UBCG) pipeline. The UBCGs are extracted using Prodigal and hmmsearch from a whole genome assembly. A set of JSON files containing UBCG sequences and metadata of the genome assemblies are selected for multiple alignments of each gene using MAFFT. The phylogenetic trees are inferred for a concatenated sequence of the 92 UBCGs (Na et al., 2018). The selection of 92 core genes is informed by a comprehensive dataset consisting of 1,429 complete genome sequences spanning 28 phyla, guaranteeing the inclusion of genes that are either widely distributed across genomes or exhibit high conservation as single-copy genes. A maximum-likelihood phylogenetic tree is constructed using RAxML under the Genetic Testing Registry (GTR) model. The value of bootstrap is 1,000 in the phylogenetic analysis. No reference genomes are used during the analysis and the phylogenetic tree is unrooted, illustrating the genetic relationships among the strains without implying an evolutionary direction.

## Results

### ST, K, and O loci diversity

**Figure 1:**
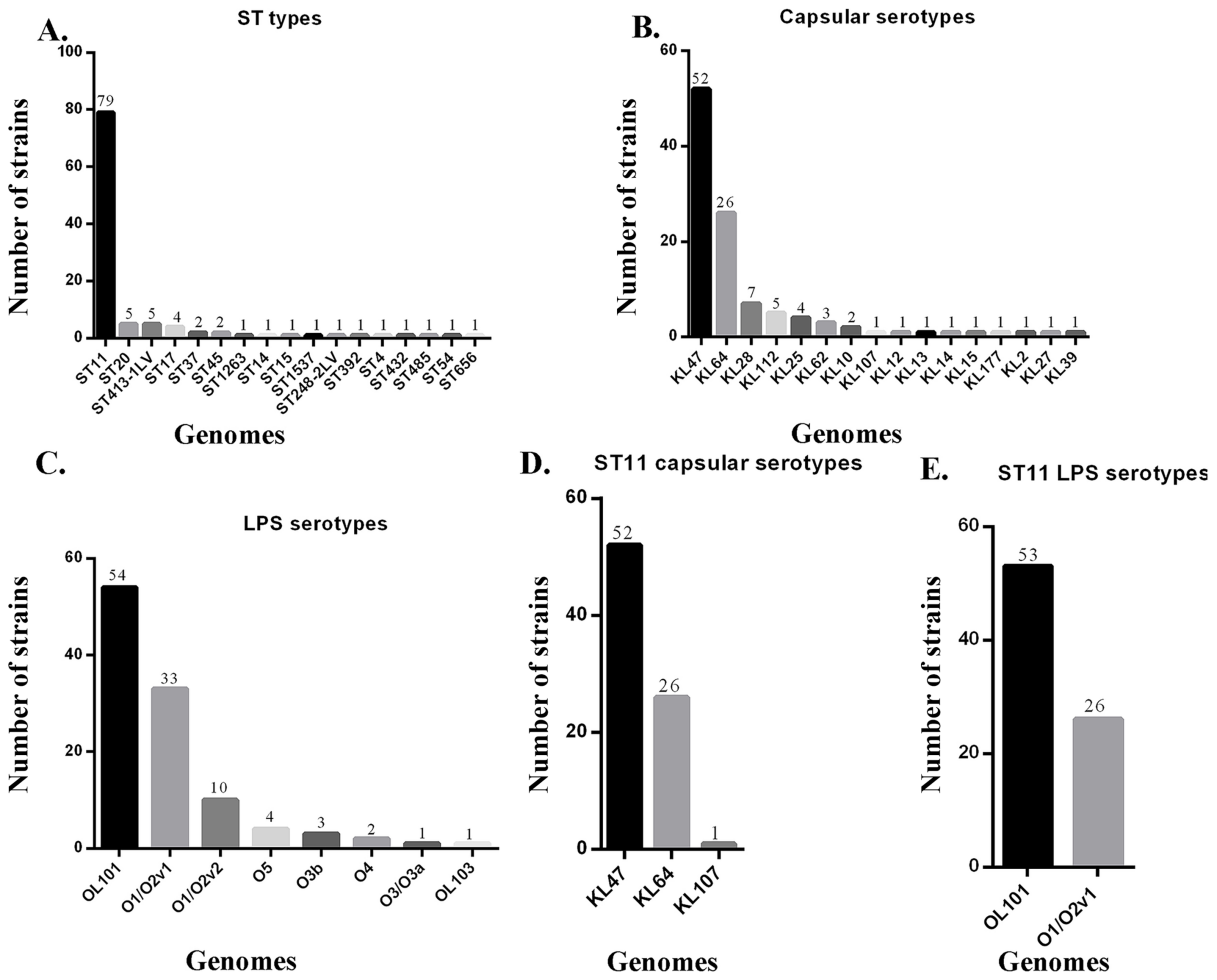
ST, K, and O loci diversity of carbapenem-resistant *K. pneumoniae*. **(A)** ST types of carbapenem-resistant *K. pneumoniae*; **(B)** Capsular serotypes of carbapenem-resistant *K. pneumoniae*; **(C)** LPS serotypes of carbapenem-resistant *K. pneumoniae*; **(D)** Capsular serotype distribution of ST11 of carbapenem-resistant *K. pneumoniae*; **(E)** LPS serotype distribution of ST11 of carbapenem-resistant *K. pneumoniae*.

**Figure 2:**
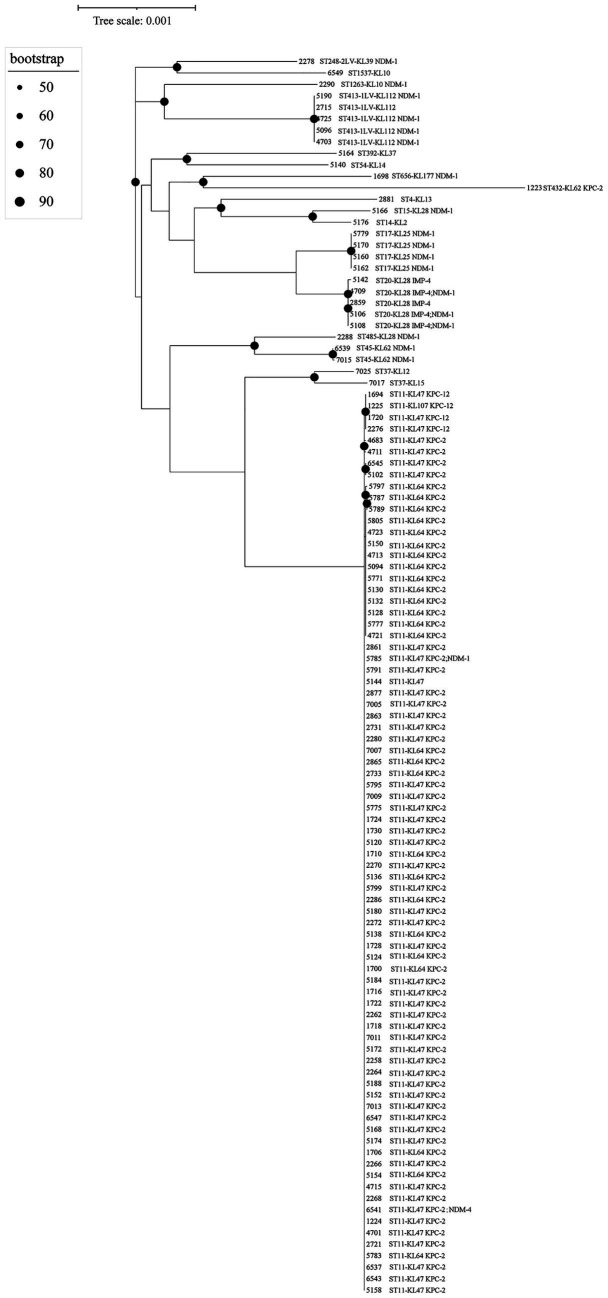
The phylogenetic tree. The extraction of core genes was carried out using the Up-to-date Bacterial Core Gene (UBCG) pipeline. Subsequently, these genes were concatenated, and a maximum-likelihood tree was constructed employing the Genetic Testing Registry (GTR) model, facilitated by the RAxML (v. 7.0.4) tool.

The sources of the 108 specimens are shown in Table 1. The main sources were sputum (34, 31.48%) and blood (26, 24.07%). Most of the specimens were from intensive care unit (ICU) wards (44, 40.74%) including neurological ICU (15, 13.89%), general ICU (14, 12.96%), medical ICU (11, 10.19%), cardiothoracic ICU (3, 2.78%), and pediatric ICU (1, 0.93%). The antimicrobial susceptibility results of strains are shown in Supplementary Table S1. The 108 CRKP isolates comprised 17 STs of CRKP, of which the dominant type was ST11 (79, 73.15%) (Table 2, Figure 1A). The evolutionary relationships of the 108 CRKP strains are shown in Figure 2. The phylogenetic tree was mainly divided into four groups: group 1 (5 strains), group 2 (4 strains), group 3 (5 strains), and group 4 (79 strains). A total of 16 K locus antigens were detected in the CRKP population, with KL47 being the most prevalent (52, 48.15%, all belong to ST11), followed by KL64 (26, 24.07%, all belong to ST11) (Table 3, Figure 1B). The most prevalent capsular serotype among ST11 isolates was KL47 followed by KL64, and these two capsular serotypes accounted for 98.73% of ST11 (Figure 1D). There were eight O locus antigens identified, with OL101 being the most frequently encountered (54, 50.00%), followed by O1/O2v1 (33, 30.56%) (Table 3, Figure 1C). The most prevalent LPS serotype of ST11 was OL101 followed by O1/O2v1; all the ST11 strains belonged to these two LPS serotypes (Figure 1E).

**Table 1:** The source of the 108 carbapenem-resistant *K. pneumoniae* species.

Specimen	*n* (%)
Sputum	34 (31.48)
Blood	26 (24.07)
Urine	13 (12.04)
Drainage fluid	11 (10.19)
Bronchoalveolar lavage fluid (BALF)	4 (3.70)
Secretion	3 (2.78)
Pus	3 (2.78)
Bile	1 (0.93)
Feces	1 (0.93)
Unknown	12 (11.11)

Wards	*n* (%)
Neurological intensive care unit (NICU)	15 (13.89)
General intensive care unit (ICU)	14 (12.96)
Medical intensive care unit (MICU)	11 (10.19)
Cardiothoracic intensive care unit (CTICU)	3 (2.78)
Pediatric intensive care unit (PICU)	1 (0.93)
Organ transplantation department	8 (7.41)
Neurology department	8 (7.41)
Pediatric surgery department	8 (7.41)
Burn surgery department	5 (4.63)
Rehabilitation medicine department	2 (1.85)
Department of gastrointestinal surgery	2 (1.85)
Department of respiratory medicine	2 (1.85)
Department of neurosurgery	2 (1.85)
Department of general internal medicine	2 (1.85)
Emergency department	2 (1.85)
Department of cardiology	2 (1.85)
Department of hepatobiliary and pancreatic surgery	2 (1.85)
Others	7 (6.48)
Unknown	12 (11.11)

**Table 2:** The ST diversity and corresponding capsular and LPS serotypes of carbapenem-resistant *K. pneumoniae*.

ST types	Capsular serotypes (*n*)	LPS serotypes (*n*)	*n* (%)
ST11	KL47 (52), KL64 (26), KL107 (1)	OL101 (53), O1/O2v1 (26)	79 (73.15%)
ST20	KL28	O1/O2v2	5 (4.63%)
ST413-1LV	KL112	O1/O2v2	5 (4.63%)
ST17	KL25	O5	4 (3.7%)
ST37	KL12 (1), KL15 (1)	O4 (1), OL103 (1)	2 (1.85%)
ST45	KL62	O1/O2v1	2 (1.85%)
ST4	KL13	O1/O2v1	1 (0.93%)
ST14	KL2	O1/O2v1	1 (0.93%)
ST15	KL28	O1/O2v1	1 (0.93%)
ST54	KL14	O3b	1 (0.93%)
ST248-2LV	KL39	O3b	1 (0.93%)
ST392	KL27	O4	1 (0.93%)
ST432	KL62	O1/O2v1	1 (0.93%)
ST485	KL28	O1/O2v1	1 (0.93%)
ST656	KL177	OL101	1 (0.93%)
ST1263	KL10	O3/O3a	1 (0.93%)
ST1537	KL10	O3b	1 (0.93%)

**Table 3:** The K and O loci diversity and corresponding ST types of carbapenem-resistant *K. pneumoniae.*

Capsular serotypes	ST types (*n*)	*n* (%)
KL47	ST11	52 (48.15%)
KL64	ST11	26 (24.07%)
KL28	ST20 (5), ST15 (1), ST485 (1)	7 (6.48%)
KL112	ST413-1LV	5 (4.63%)
KL25	ST17	4 (3.70%)
KL62	ST45 (2), ST432 (1)	3 (2.87%)
KL10	ST1263 (1), ST1537 (1)	2 (1.85%)
KL2	ST14	1 (0.93%)
KL12	ST37	1 (0.93%)
KL13	ST4	1 (0.93%)
KL14	ST54	1 (0.93%)
KL15	ST37	1 (0.93%)
KL27	ST392	1 (0.93%)
KL39	ST248-2LV	1 (0.93%)
KL107	ST11	1 (0.93%)
KL177	ST656	1 (0.93%)

LPS serotypes	ST types (*n*)	*n* (%)
OL101	ST11 (53), ST656 (1)	54 (50.00%)
O1/O2v1	ST11 (26), other STs (7)	33 (30.56%)
O1/O2v2	ST20 (5), ST413-1LV (5)	10 (9.26%)
O5	ST17	4 (3.70%)
O3b	ST54 (1), ST248-2LV (1), ST1537 (1)	3 (2.78%)
O4	ST392 (1), ST37 (1)	2 (1.85%)
O3/O3a	ST1263	1 (0.93%)
OL103	ST37	1 (0.93%)

### Distribution of acquired resistance genes by antibiotic class

**Figure 3:**
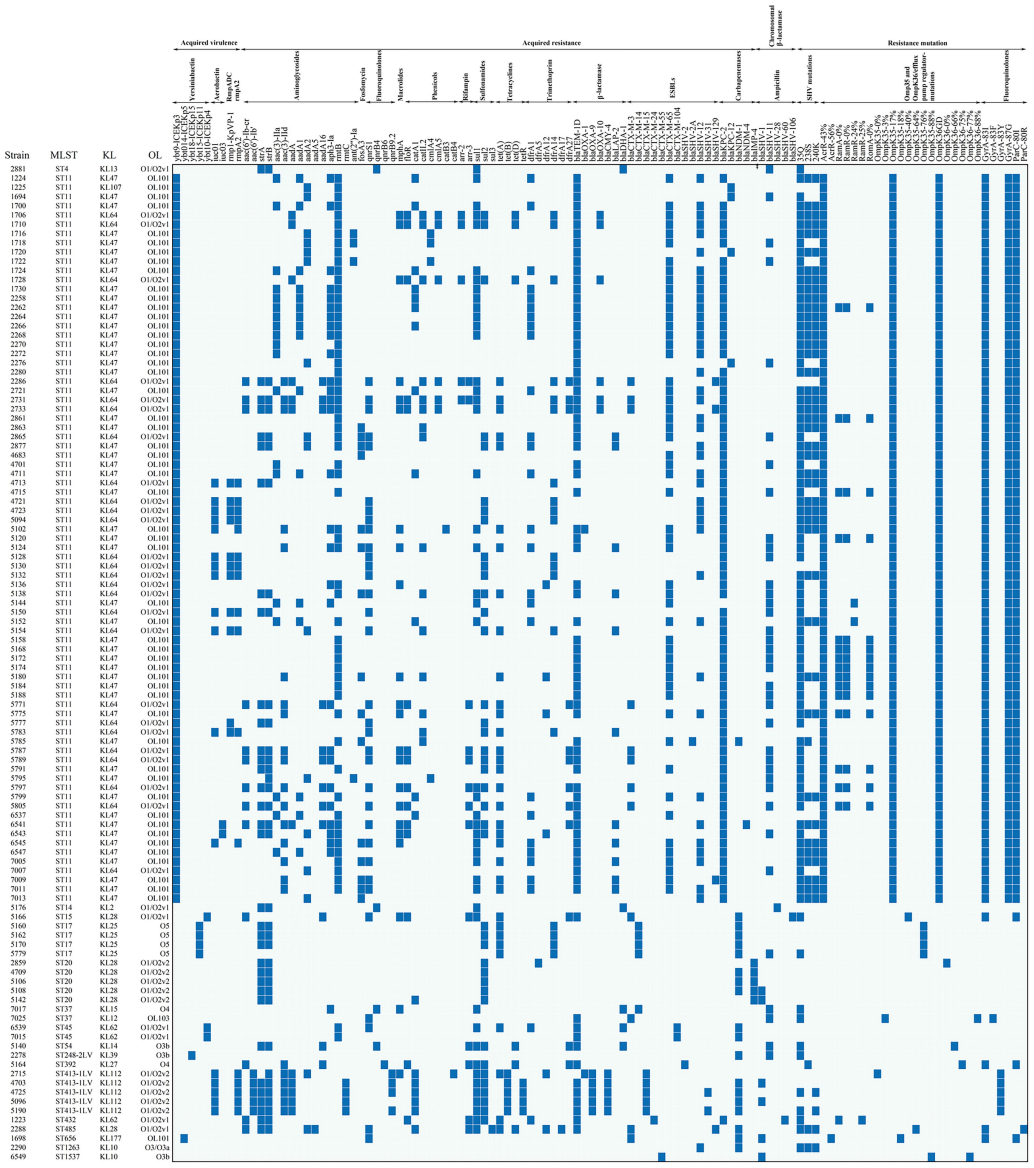
Distribution of acquired resistance genes by antibiotic class, resistance mutation, and acquired virulence factor. Each small blue square indicates the presence of acquired resistance genes, resistance mutations, and virulence factors.

**Figure 4:**
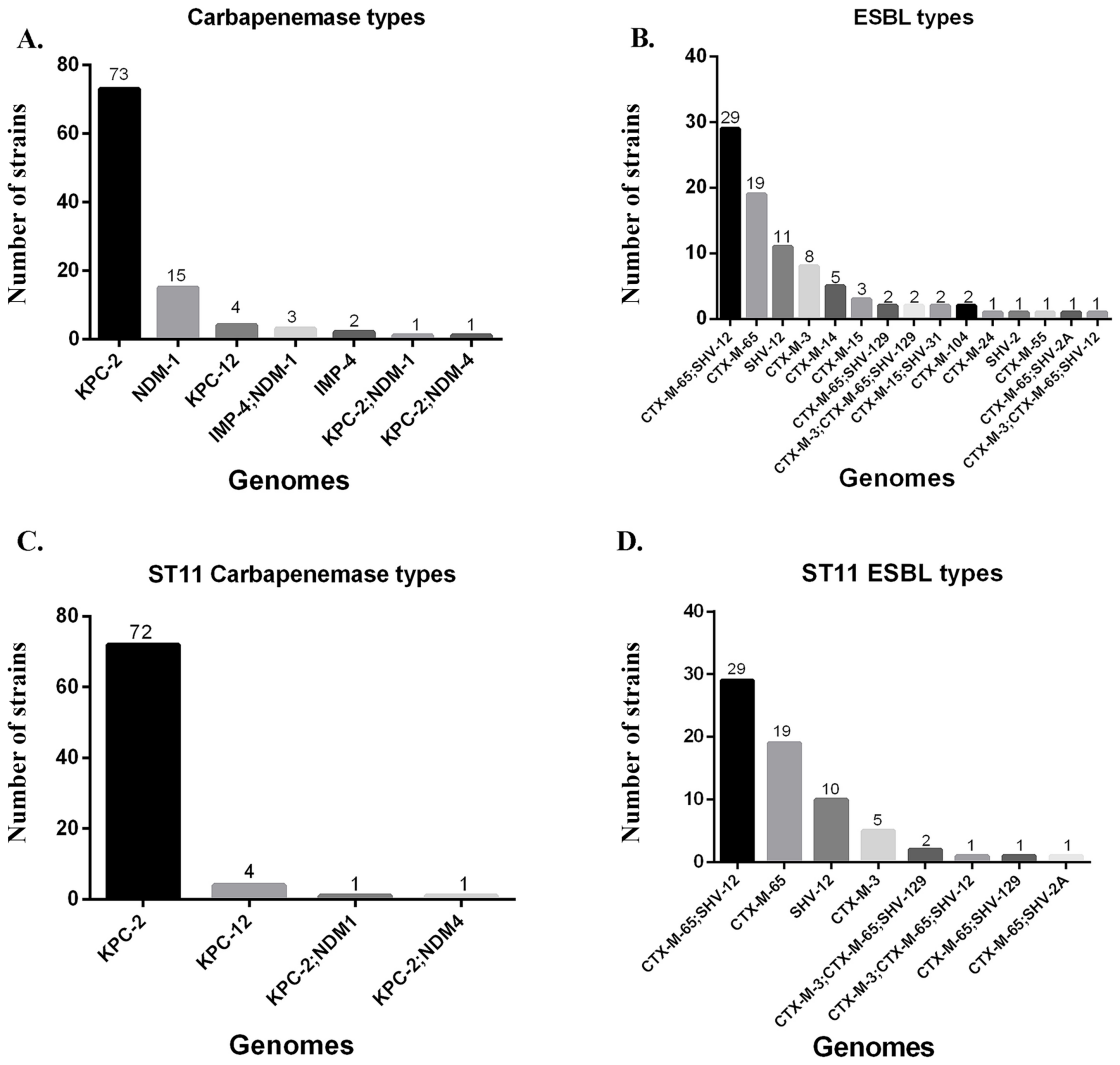
Carbapenemase and extended-spectrum β-lactamase types of carbapenem-resistant *K. pneumoniae*. **(A)** Carbapenemase types of carbapenem-resistant *K. pneumoniae*; **(B)** Extended-spectrum β-lactamase types of carbapenem-resistant *K. pneumoniae*; **(C)** Carbapenemase distribution of ST11 carbapenem-resistant *K. pneumoniae*; **(D)** Extended-spectrum β-lactamase distribution of ST11 carbapenem-resistant *K. pneumoniae*.

68 acquired AMR genes spanning 12 antibiotic classes were detected in the CRKP population (Table 4, Figure 3). Among these CRKP, KPC-2 (73, 67.59%) was the dominant carbapenemase type, followed by NDM-1 (15, 13.89%) (Figure 4A). Furthermore, our study identified four CRKP strains producing KPC-12, which is a rarely reported KPC-2 variant that has not previously been documented in South China. In addition, a few strains carried combinations of carbapenemases, including KPC-2 and NDM-1 (1, 0.93%) and IMP-4 and NDM-1 (3, 2.78%). Six strains exhibited mutations of porins in the absence of acquired carbapenemase genes, specifically involving OmpK35 only (*n* = 1), OmpK36 only (*n* = 1), and both OmpK35 and OmpK36 (*n* = 4). Three strains were resistant to carbapenems despite lacking carbapenemase genes and carrying only lactamases. KPC-2 was mainly associated with ST11, while NDM-1 had ST413-1LV and ST17 as the dominant clonotypes. All the strains harboring IMP-4 and co-harboring IMP-4 and NDM-1 belonged to ST20 and all KPC-12 isolates belonged to ST11. The main carbapenemase types of ST11 was KPC-2 followed by KPC-12, co-carriage of KPC-2 and NDM-1, and co-carriage of KPC-2 and NDM-4. (Figure 4C). The most commonly identified ESBL was co-carriage of CTX-M-65 and SHV-12 (29, 26.85%), followed by CTX-M-65 (19, 17.59%), SHV-12 (11, 10.19%), CTX-M-3 (8, 7.41%), CTX-M-14 (5, 4.63%), and CTX-M-15 (3, 2.78%) (Figure 4B), together accounting for 85.23% of all ESBLs. The main ESBL types of ST11 CRKP included co-carriage of CTX-M-65 and SHV-12, as well as CTX-M-65 alone and SHV-12 alone (Figure 4D).

**Table 4:** Antibiotic classes associated with the acquired AMR genes harbored by the carbapenem-resistant *K. pneumoniae*.

Antibiotic classes	*n* (%)
Carbapenems	99 (91.67%)
Aminoglycosides	94 (87.04%)
Extended-spectrum Β-lactamase (ESBL)	88 (81.48%)
β-lactamase	82 (75.93%)
Sulfonamides	71 (65.74%)
Trimethoprim	62 (57.41%)
Phenicols	50 (46.30%)
Fluoroquinolones	42 (38.89%)
Tetracyclines	42 (38.89%)
Macrolides	22 (20.37%)
Rifampin	16 (14.81%)
Fosfomycin	15 (13.89%)
Colistin	Not detected
Clycopeptides	Not detected
Tigecycline	Not detected
β-lactamase inhibitor	Not detected
ESBL inhibitor	Not detected

Acquired β-lactamase genes included *bla*_TEM-1D_, *bla*_OXA-1_, *bla*_OXA-9_, *bla*_OXA-10_, *bla*_CMY-4_, *bla*_LAP-2_, and *bla*_DHA-1_, with *bla*_TEM-1D_ (76, 70.37%) being the most prevalent. There were 15 aminoglycoside acquired genes and *rmtB* (61, 56.48%), *trB* (36, 33.33%) *strA* (34, 31.48%), and *aph3-Ia* (29, 26.85%) were the most frequent. The main acquired genes conferring resistance to sulfonamides, tetracyclines, and fluoroquinolones were *sul1* and *sul2* (70, 64.81%), *tet(A)* (33, 30.56%), and *qnrS1* (33, 30.56%), respectively. The acquired trimethoprim resistance genes mainly consisted of *dfrA1* (27, 25%) and *dfrA14* (19, 17.59%). The acquired phenicol genes mainly included *catA1* (19, 17.59%), *floR* (15, 13.89%), and *catII* (15, 13.89%). Acquired resistance genes for fosfomycin, macrolides, and rifampin were *fosA3*, *mphA*, and *arr-2* and *arr-3*, respectively. Among the 108 CRKP strains, the intrinsic chromosomal β-lactamases (SHVs) occurred in 42.59% (46/108) (Figure 3).

### Chromosomal-encoded mutations

Amino acid substitutions Q35, S238, and K240 in the intrinsic SHV β-lactamase were identified in these strains. Among the 108 CRKP, the prevalence of 35Q, 238S, and 240K mutations was 79.63, 42.59, and 42.59%, respectively. Moreover, 44 (40.78%) strains carried 35Q, 238S, and 240K at the same time. Among the 108 CRKP strains, 86.11% (93/108) had mutations in OmpK35, OmpK36 porins, and efflux pump regulators, and a total of 21 distinct mutations had been detected. 64 (59.26%) strains possessed mutations with AcrR-43%, OmpK35-17%, and OmpK36GD simultaneously. As for the fluoroquinolones mutations, 82.40% (89/108) strains had mutations in GyrA (positions 83 and 87) and ParC (positions 80 and 84). 73.15% (79/108) strains had a mutation combination of GyrA-83I, GyrA-87G, and ParC-80I. All the ST11 CRKP strains in our study had porin and efflux pump mutations of AcrR-43%, OmpK35-17%, and OmpK36GD and fluoroquinolones mutations of GyrA-83I, GyrA-87G, and ParC-80I. 53.16% (42/79) ST11 CRKP strains in our study had SHV β-lactamase mutations of 35Q, 238S, and 240K (Figure 3).

### Virulence

**Figure 5:**
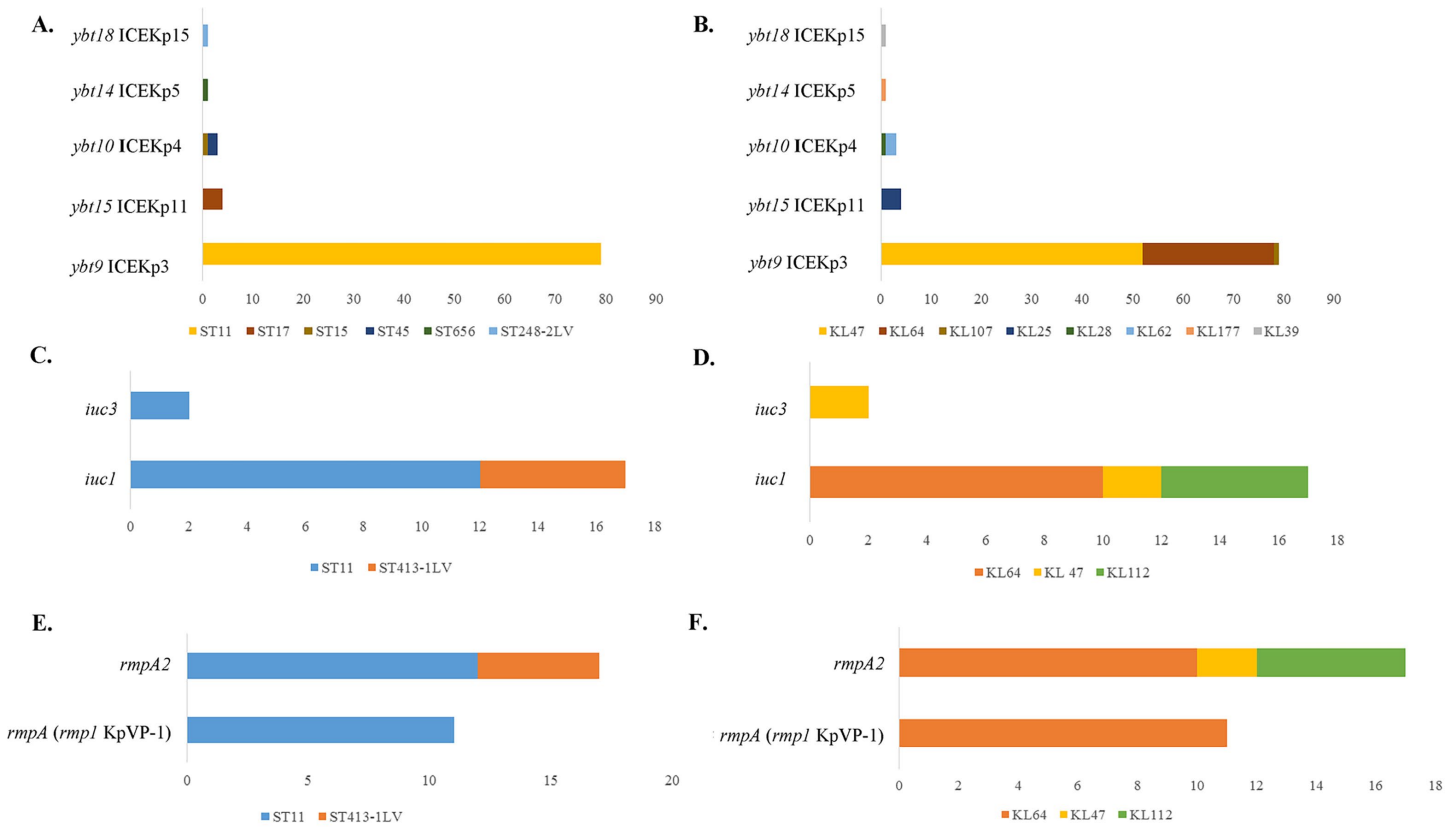
Distribution of virulence genes among carbapenem-resistant *K. pneumoniae* of different STs and capsular serotypes. **(A)** Distribution of yersiniabactin among CRKP of different STs; **(B)** Distribution of yersiniabactin among CRKP of different capsular serotypes; **(C)** Distribution of aerobactin among CRKP of different STs; **(D)** Distribution of aerobactin among CRKP of different capsular serotypes; **(E)** Distribution of *rmpA* and *rmpA2* among CRKP of different STs; **(F)** Distribution of *rmpA* and *rmpA2* among CRKP of different capsular serotypes.

*ybt* had been detected in 81.48% (88/108) of the strains, with *ybt9* ICEKp3 (79, 73.15%) the most common lineage, followed by *ybt15* ICEKp11 lineage (4, 3.70%), *ybt10* ICEKp4 lineage (3, 2.78%), *ybt14* ICEKp5 lineage (1, 0.93%), and *ybt18* ICEKp15 lineage (1, 0.93%) (Figure 5A). All the ST11 CRKP isolates contained *ybt9* ICEKp3 lineages. 52 KL47 CRKP isolates, 26 KL64 CRKP isolates, and 1 KL107 CRKP isolate had *ybt9* ICEKp3 lineages. The four ST17-KL25 CRKP isolates contained *ybt15* ICEKp11 lineages (Figures 5A,B). *iuc* was identified in 17.59% (19/108) of the CRKP isolates, with a detection rate of 15.74% (17/108) for *iuc1* lineage and 1.85% (2/108) for *iuc3* lineage (Figure 5C). The 17 CRKP isolates carrying *iuc1* lineages included 12 ST11 CRKP isolates and five ST413-1LV CRKP isolates. The 12 ST11 CRKP isolates contained 10 KL64 and two KL47 isolates, and five ST413-1LV CRKP isolates all belonged to KL112. The two CRKP isolates carrying *iuc3* lineages both belonged to ST11 and KL47 (Figures 5C,D).

The detection rates of rmpA and rmpA2 were 10.19% (11/108) and 15.74% (17/108), respectively. The 11 *rmpA* positive strains contained *rmp1* KpVP-1 and belonged to ST11 and KL64 (Figures 5E,F). The detected results for *rmpA2* were similar to those of *iuc*. The 17 *rmpA2* positive strains included 12 ST11 and 5 ST413-1LV CRKP isolates. The 12 ST11 CRKP isolates contained 10 KL64 and two KL47 isolates. The five ST413-1LV CRKP isolates all belonged to KL112 (Figures 5E,F). Among the 108 CRKP strains in our study, *clb* and *iro* were not been detected. What’s more, there were 17 hvCRKP (17/108, 15.74%) detected in our study, including 10 ST11-KL64 hvCRKP positive for *rmpA*, *rmpA2*, and *iuc1* (Table 5). Seven hvCRKP were *rmpA2* and *iuc1* positive, including five ST413-1LV-KL112 and 2 ST11-KL47 strains. The 10 ST11-KL64 and two ST11-KL47 hvCRKP all produced KPC-2 and had mutations of AcrR-43%, OmpK35-17%, and OmpK36GD. The five ST413-1LV-KL112 hvCRKP included four NDM-1-producing strains and one non-carbapenemase-producing strain. The ST413-1LV-KL112 hvCRKP producing NDM-1 is a new ST type among hvCRKP-producing NDM-1 strains and has not been reported in other reports before, indicating ST413-1LV-KL112 hvCRKP-producing NDM-1 may be a reginal specific isolate in South China and a new challenge to clinical therapy. Moreover, the four ST413-1LV-KL112 hvCRKP harboring the *bla*_NDM-1_ gene also contained the ESBL gene *bla*_CTX-M-15_ and β-lactamase genes including *bla*_CMY-4_, *bla*_OXA-9_, and *bla*_TEM-1D_. The ST413-1LV-KL112 hvCRKP without *bla*_NDM-1_ gene contained the ESBL gene *bla*_CTX-M-15_ and β-lactamase genes including *bla*_CMY-4_, *bla*_OXA-1_, and *bla*_OXA-9_.

**Table 5:** Characteristics of hypervirulent carbapenem-resistant *K. pneumoniae.*

Strain	ST	Serotype	carbapenemase	OmpK35 and OmpK36/efflux pump regulator mutations	ESBL	β-lactamase	Aerobactin (*iuc* Lineage)	Yersiniabactin (*ybt* Lineage)	*RmpA* (*rmp* Lineage)/*RmpA2*
4713	ST11	KL64	KPC-2	AcrR-43%; OmpK35-17%; OmpK36GD	SHV-12	/	*iuc1*	*ybt9* ICEKp3	*rmp1* KpVP-1; *rmpA2*
4721	ST11	KL64	KPC-2	AcrR-43%; OmpK35-17%; OmpK36GD	SHV-12	/	*iuc1*	*ybt9* ICEKp3	*rmp1* KpVP-1; *rmpA2*
4723	ST11	KL64	KPC-2	AcrR-43%; OmpK35-17%; OmpK36GD	SHV-12	/	*iuc1*	*ybt9* ICEKp3	*rmp1* KpVP-1; *rmpA2*
5094	ST11	KL64	KPC-2	AcrR-43%; OmpK35-17%; OmpK36GD	SHV-12	/	*iuc1*	*ybt9* ICEKp3	*rmp1* KpVP-1; *rmpA2*
5128	ST11	KL64	KPC-2	AcrR-43%; OmpK35-17%; OmpK36GD	/	/	*iuc1*	*ybt9* ICEKp3	*rmp1* KpVP-1; *rmpA2*
5130	ST11	KL64	KPC-2	AcrR-43%; OmpK35-17%; OmpK36GD	/	/	*iuc1*	*ybt9* ICEKp3	*rmp1* KpVP-1; *rmpA2*
5132	ST11	KL64	KPC-2	AcrR-43%; OmpK35-17%; OmpK36GD	SHV-12	/	*iuc1*	*ybt9* ICEKp3	*rmp1* KpVP-1; *rmpA2*
5150	ST11	KL64	KPC-2	AcrR-43%; OmpK35-17%; OmpK36GD	/	/	*iuc1*	*ybt9* ICEKp3	*rmp1* KpVP-1; *rmpA2*
5154	ST11	KL64	KPC-2	AcrR-43%; OmpK35-17%; OmpK36GD	/	LAP-2	*iuc1*	*ybt9* ICEKp3	*rmp1* KpVP-1; *rmpA2*
5783	ST11	KL64	KPC-2	AcrR-43%; OmpK35-17%; OmpK36GD	/	LAP-2	*iuc1*	*ybt9* ICEKp3	*rmp1* KpVP-1; *rmpA2*
5102	ST11	KL47	KPC-2	AcrR-43%; OmpK35-17%; OmpK36GD	CTX-M-65; SHV-12	OXA-1; TEM-1D	*iuc 1*	*ybt9* ICEKp3	*rmpA2*
6545	ST11	KL47	KPC-2	AcrR-43%; OmpK35-17%; OmpK36GD	CTX-M-65; SHV-12	LAP-2; TEM-1D	*iuc1*	*ybt9* ICEKp3	*rmpA2*
2715	ST413-1LV	KL112	/	OmpK35-0%	CTX-M-15	CMY-4; OXA-1; OXA-9	*iuc1*	/	*rmpA2*
4703	ST413-1LV	KL112	NDM-1	/	CTX-M-15	CMY-4; OXA-9; TEM-1D	*iuc1*	/	*rmpA2*
4725	ST413-1LV	KL112	NDM-1	/	CTX-M-15; SHV-31	CMY-4; OXA-9; TEM-1D	*iuc1*	/	*rmpA2*
5096	ST413-1LV	KL112	NDM-1	/	CTX-M-15	CMY-4; OXA-9; TEM-1D	*iuc1*	/	*rmpA2*
5190	ST413-1LV	KL112	NDM-1	/	CTX-M-15; SHV-31	CMY-4; OXA-9; TEM-1D	*iuc1*	/	*rmpA2*

## Discussion

CRKP has been designated by the WHO as a critical priority pathogen because of its high fatality rate and limited options available for targeted therapy (Zhu et al., 2024). It has been steadily disseminating worldwide and poses challenges to clinical therapy. Recently, CRKP has undergone various changes in transmission dynamics, antimicrobial resistance, and virulence. In our study, we aimed to explore the molecular epidemiology of antimicrobial resistance and virulence in CRKP in South China to provide insights into CRKP regional epidemic patterns, guiding individualized antibacterial therapy and developing vaccine targets. In our research, the dominant ST was ST11, accounting for 73.15% of all the strains, followed by ST20, ST413-1LV, and ST17. The predominant carbapenemase was KPC-2, with 91.67% of the strains carrying the *bla*_KPC-2_ gene. Meanwhile, KPC-2 was predominantly associated with ST11 (72/73, 98.63%), indicating a high prevalence of ST11 CRKP carrying the *bla*_KPC-2_ gene. In a systematic review and meta-analysis on the resistance profile of CRKP including 68 studies from 19 provinces in China, the most prevalent CRKP-associated STs were ST11, followed by ST15 and ST20, and the predominant carbapenemase type was KPC-2, followed by NDM-1 (Yang et al., 2025). In China, ST11 is consistently the dominant lineage across different regions, while the distribution of other sequence types exhibits regional variation.

Moreover, four CRKP isolates producing KPC-12 were identified in our study, including three ST11-KL47 isolates and one ST11-KL107 isolate. KPC-12 is a newly discovered and rarely reported variant of KPC-2, characterized by an L169M substitution in the *Ω* loop (Qin et al., 2021). In the National Center for Biotechnology Information^2^ database, there are currently only four KPC-12 variants uploaded: two from East China and two from West China. The four KPC-12 variants identified in South China appear to be region-specific KPC-2 derivatives. These findings address a research gap regarding KPC-12 in South China and provide a foundation for studying such variants nationwide. Recently, novel β-lactam/β-lactamase inhibitor combinations, such as ceftazidime-avibactam (CZA), meropenem-vaborbactam, and imipenem-relebactam, have been developed to address infections caused by carbapenemase-producing Enterobacterales. CZA displays potent *in vitro* activity against KPC-producing Enterobacterales and is considered one of the best treatment options for infections caused by KPC-producing strains, particularly *K. pneumoniae* (Yin et al., 2019). One of the most important phenotypic traits of KPC variants including KPC-12 is their resistance to CZA. KPC variants could mediate bacterial resistance to CZA by changing KPC structure to enhance the affinity to ceftazidime and weaken the affinity to avibactam (Humphries et al., 2015). Moreover, to date, no guidelines or expert consensus have been established to inform the diagnosis and management of infections caused by KPC variants (Falcone et al., 2026). The emergence of KPC-12 indicates rapid resistance among CRKP in South China. Owing to its resistance to CZA, the KPC-12 variant can necessitate a shift in clinical therapeutic strategies from CZA to other novel β-lactam/β-lactamase inhibitor combinations, tigecycline, or polymyxin and heighten the urgency for new drug development. Furthermore, in the CRKP ST11-KL47 clonal background, strains carrying *bla*_KPC-12_ are more likely to exhibit a CZA-resistance phenotype (Han et al., 2024). The patients infected with CRKP producing KPC-12 in our study died of multiple bacterial infections or underwent organ resection. Therefore, KPC-12 warrants particular attention in South China, and its early and accurate identification is essential. Moreover, a novel KPC variant, KPC-204, which confers resistance to both carbapenems and CZA in ST11 CRKP, has been identified in West China (Gong et al., 2024). Five KPC-33 and three KPC-93 variants among 477 CRKP isolates from 46 hospitals in Zhejiang Province from 2018 to 2021 have been detected (Liu et al., 2022). Subsequent studies have reported the emergence of other KPC variants nationwide, such as KPC-155, KPC-185, KPC-204, KPC-207, and KPC-228 (Tang et al., 2024; Guo et al., 2025; Zhou et al., 2025). The identification of diverse KPC variants nationwide emphasizes the need for vigilant monitoring, underscoring the importance of strengthening routine detection and reporting in clinical microbiology laboratories, as well as developing methods that can quickly and accurately identify novel KPC variants.

In this study, KL47 was the most prevalent capsular serotype, followed by KL64. Together, these two serotypes accounted for 72.22% of all CRKP isolates. Among ST11 strains, KL47 and KL64 were the predominant capsular serotypes. Recently, the population of ST11-KL64 has increased rapidly and gradually surpassed that of ST11-KL47 in China (Shi et al., 2024). However, even though ST11-KL64 accounts for a proportion of CRKP, ST11-KL47 remains the dominant clone in South China. Regarding LPS serotypes, OL101 was the most prevalent, followed by O1/O2v1. Together, these two types accounted for 80.56% of all CRKP isolates. In addition to the predominant types, other KL types (e.g., KL28, KL112, and KL25) and OL types (e.g., O1/O2v2, O5, and O3b) were also identified. Understanding the regional distribution patterns of KL and OL is important for surveillance, interventions, and targeted vaccine strategies to handle CRKP infection in South China.

OmpK35 and OmpK36 were two main porins of *K. pneumoniae* that could regulate antibiotic influx across the outer membrane (Gaibani et al., 2022). Porin mutations, particularly when combined with carbapenemase production, can result in high levels of resistance. *acrR* is known as the major transcriptional repressor of the main multidrug efflux pump in Enterobacteriaceae (Maldonado et al., 2023). Mutations in *acrR* can confer resistance through regulation of the AcrAB-TolC efflux pump (Worley et al., 2023). The ram locus, comprising *romA*-*ramA*, is negatively regulated by *ramR*, while *ramA* functions as a global transcriptional activator (De Majumdar et al., 2014). Mutations in *ramR* and the *ramR-ramA* intergenic region of *K. pneumoniae* can lead to increased expression of *ramA*, enhancing efflux-mediated multidrug resistance (Yamasaki et al., 2013). In our study, the most prevalent porin mutations were OmpK35-17% and OmpK36GD, while the most prevalent efflux pump regulator mutation was AcrR-43%. All the ST11 CRKP in our study harbored porin mutations (OmpK35-17% and OmpK36GD), an efflux pump regulator mutation (AcrR-43%), and fluoroquinolones mutations (GyrA-83I, GyrA-87G, ParC-80I), which was consistent with recent related reports. It has been reported that ST11 CRKP strains with OmpK35-del and OmpK36GD exhibited higher MICs to carbapenems and OmpK35-17% and OmpK36GD were the most prevalent in ST11 CRKP in China (Qin et al., 2026). In a molecular epidemiological analysis of CRKP in China, OmpK35-17% and OmpK36GD were also the most common porin mutations, and 89.4% of the isolates carrying the mutations in the study belonged to ST11 (Zhao et al., 2022). The successful dissemination of ST11 CRKP may be related to some crucial evolutionary factors: acquisition of the KPC-2 plasmid, alterations in OmpK35/OmpK36 and efflux pump regulators leading to carbapenem resistance, fluoroquinolones mutations, and acquisition of virulence plasmids. According to the WGS results, all 108 CRKP strains exhibited complex MDR phenotypes and carried MDR genes, including ESBL, β-lactamase, aminoglycoside, sulfonamide, trimethoprim, phenicol, fluoroquinolone, and tetracycline, indicating increasing prevalence of MDR CRKP. In addition to carbapenem resistance, OmpK35/OmpK36 and efflux pump regulator mutations could mediate the resistance to multiple antibiotics including cephalosporins, tigecycline, fluoroquinolones, and aminoglycosides (Ejaz, 2022; Park et al., 2025). The coexistence of multiple drug-resistant genes and porin and efflux pump regulator mutations would further enhance the level of antibiotic resistance, making it crucial to take multilevel, high-intensity, and regionally coordinated interventions to control the spread of MDR CRKP.

Three major virulence factors, including *ybt*, *iuc*, and *rmpA/rmpA2*, were detected among the 108 CRKP isolates. ICEKp is an integrative conjugative element of *K.pneumoniae,* and *ybt* locus is frequently embedded in ICEKp (Jati et al., 2023). Our data showed 73.15% of CRKP isolates carried *ybt 9* ICEKp3, and all ST11 isolates harbored this lineage, indicating its high prevalence, especially in ST11 CRKP. In the Kleborate virulence scoring system, the presence of *ybt*, *clb*, and *iuc* is used to assign a virulence score as follows: 0 = none present, 1 = *ybt* only, 2 = *clb* without *iuc* (regardless of *ybt*), 3 = *iuc* only, 4 = *iuc* and *ybt* without *clb*, and 5 = all three present. The hvCRKP corresponds to a virulence score ≥3 (carrying *iuc*). In the scoring system, no direct scores are assigned to *rmpA*/*rmpA2*, which are key regulators of the hypermucoviscous phenotype related to strain virulence. The most obvious phenotypic feature of hvKp is hypermucoviscous (Choby et al., 2020). Compared with the Kleborate virulence scoring system, our criteria for hvCRKP isolates are more stringent; we require the presence of both iuc and the rmpA/rmpA2 genes associated with the hypermucoviscous phenotype. A recent study evaluating markers of hypervirulence demonstrated that the genes *peg-344*, *iroB*, *iucA*, *rmpA*, and *rmpA2* all demonstrated >0.95 diagnostic accuracy for identifying hvKp strains, and these markers were regarded as highly diagnostic of hypervirulence (Russo et al., 2018). Thus, many researchers have adopted the presence of some of these markers to classify a strain as being hypervirulent, most notably a combination of *iuc* and *rmpA*/*rmpA2* (Russo et al., *2024*). In Anton Spadar’s study, strains were identified as hvKp if they contained either *iuc*/*iro* or both gene loci (Spadar et al., 2023). In Yi Wang’s research, isolates with at least one of the genes *peg-344*, *iroB*, *iucA*, *rmpA*, and *rmpA2* were classified as hvKp (Wang Y. et al., 2022). While in Danni Pu’s, Ferreira Raro’s, and Fatma’s research, strains carrying *iuc* and *rmpA*/*rmpA2* were identified as hvKp (Hallal Ferreira Raro et al., 2023; Pu et al., 2023; Mohamed et al., 2025). Therefore, in our article, we selected the more commonly used and highly accurate hvKp interpretive criteria. I*uc* and *rmpA/rmpA2* are two essential virulence factors for hvCRKP. hvCRKP has been documented in many countries and regions across the globe as having a high mortality, triggering a major public health challenge (Wyres et al., 2020). In this study, 17 isolates, mainly belonging to ST11 and ST413-1LV, exhibited both carbapenem resistance and hypervirulence-associated genes. All 12 ST11 hvCRKP isolates (10 KL64 and 2 KL47) harbored the *bla*_KPC-2_ gene, suggesting ST11-KL64 carrying *bla*_KPC-2_ is the predominant hvCRKP clone, consistent with previous studies (Wu et al., 2024). Furthermore, infections in 12 patients with hvCRKP were successfully controlled with timely and appropriate antimicrobial therapy, highlighting the importance of surveillance and early detection.

To date, most hvCRKP strains reported in China produce KPC-2, whereas NDM-1-producing hvCRKP isolates are less frequently reported (Yang et al., 2022). A study by Gu et al. reported that the most common NDM-1-producing hvCRKP type was ST23-KL1, while less frequent types included ST147-KL64, ST218-KL57, and ST17-KL112 (Gu et al., 2025). The eight hvCRKP isolates in Wang’s J. et al. (2022) study all belonged to ST23. Most NDM-1-producing hvCRKP isolates identified to date belong to ST23. However, in our study, the four hvCRKP isolates carrying the *bla*_NDM-1_ gene all belonged to ST413-1LV-KL112, which has not been reported before, indicating an emerging region-specific high-risk clone transmission of this type in South China. The concurrent presence of the *bla*_NDM-1_ gene and hypervirulence markers including *rmpA2* and *iuc1* indicates that these strains possess both enhanced pathogenicity and transmissible resistance. Moreover, the localization of *bla*_NDM-1_ on transferable plasmids facilitates horizontal gene transfer to other Enterobacteriaceae species, increasing the risk of widespread dissemination in South China. The emergence of this rare type hvCRKP could also help us understand the molecular dissemination mechanisms of NDM-1 among different hvCRKP STs in South China and the evolution of region-specific hvCRKP strains. In addition to the *bla*_NDM-1_ gene, all four ST413-1LV-KL112 hvCRKP also carried ESBL gene *bla*_CTX-M-15_ and β-lactamase genes including *bla*_CMY-4_, *bla*_OXA-9_, and *bla*_TEM-1D_, warranting particular attention. Thus, this new type of hvCRKP may change the existing treatment strategies, which should be explored further. Facing the emergence of KPC variants and the new type of hvCRKP carrying *bla*_NDM-1_, a nationwide collaborative surveillance network for KPC variants and novel hvCRKP strains carrying different carbapenem resistance genes should be established to monitor the spread of these strains in real-time. Meanwhile, continuous genomic surveillance is essential for monitoring the emergence and spread of novel KPC variants and high-risk hvCRKP strains, and rational use of antimicrobials should be advocated to avoid their emergence and dissemination.

Several limitations of our study should be acknowledged. First, though our study provided regional epidemiological features of CRKP in South China, it is mainly descriptive. Moreover, although whole-genome sequencing was performed, the analysis remains relatively basic and deeper genomic analysis including genomic context of resistance genes and plasmid structures is needed. In the follow-up research, deeper genomic analysis would be analyzed, more information about transmission dynamics would be collected, and evolutionary analysis of dominant clones would be explored, enhancing the depth of the manuscript. Second, strains carrying *iuc* and *rmpA*/*rmpA2* were identified as hvKp in our study. However, this classification is based on genomic prediction rather than phenotypic confirmation. Experimental validation of the hypervirulent phenotype would be conducted in the future to confirm our findings.

## Conclusion

In our study, the regional epidemiological features of CRKP in South China were explored. The main ST type, capsular serotype, and LPS serotype were ST11, KL47, and OL101, respectively. KPC-2 was the most prevalent carbapenemase of CRKP. Four KPC-12-producing CRKP isolates were identified, and this rare variant of KPC-2 warrants attention in South China. Moreover, all CRKP strains carried multiple drug-resistant genes and exhibited complex MDR phenotypes, posing challenges for clinical treatment. Apart from producing carbapenemase, most CRKP isolates harbored mutations in OmpK35 and OmpK36 porins and efflux pump regulators. Notably, all ST11 isolates carried mutations of AcrR-43%, OmpK35-17%, and OmpK36GD at the same time, which could further enhance antimicrobial resistance. Regarding virulence factors, the most common type of *iuc* and *ybt* were *iuc1* and *ybt9* ICEKp3, respectively. The 17 hvCRKP isolates were mainly ST11-KL64 and ST413-1LV-KL112, which harbored *bla*_KPC-2_ and *bla*_NDM-1_ genes, respectively. ST413-1LV-KL112, as a new type of hvCRKP producing NDM-1, needs further investigation. In South China, the dominant CRKP strains were ST11, KL47, and OL101, producing KPC-2 and carrying *iuc1* and *ybt9* ICEKp3, accompanied by alterations in OmpK35/OmpK36 and efflux pump regulators, as well as multiple resistance genes. Additionally, the emergence of the KPC-12 variant and the identification of a novel NDM-1-producing hvCRKP (ST) characterize the regional epidemiology of CRKP. Meanwhile, highly virulent and MDR CRKP strains are trending toward epidemic, highlighting the need for ongoing genomic surveillance and strict infection control measures.

## Data Availability

The datasets generated for this study can be found in the National Center for Biotechnology Information (https://www.ncbi.nlm.nih.gov/) under Bioproject PRJNA1450056 with Sample IDs SAMN57142711–SAMN57142818.

## Footnotes

1
https://github.com/klebgenomics/Kleborate

2
https://www.ncbi.nlm.nih.gov/
